# On the early stages of localised atmospheric corrosion of magnesium–aluminium alloys

**DOI:** 10.1038/s41598-020-78030-w

**Published:** 2020-12-01

**Authors:** M. Shahabi-Navid, Y. Cao, J. E. Svensson, A. Allanore, N. Birbilis, L. G. Johansson, M. Esmaily

**Affiliations:** 1 Engineering Quality, Powertrain Engineering, Volvo Group Trucks Technology, Gothenburg, Sweden; 2 Department of Industrial and Materials Science, Chalmers University of Technology, Gothenburg, Sweden; 3 Department of Chemistry and Chemical Engineering, Chalmers University of Technology, Gothenburg, Sweden; 4 Department of Materials Science and Engineering, Massachusetts Institute of Technology, Cambridge, USA; 5 College of Engineering and Computer Science, The Australian National University, Canberra, Australia

**Keywords:** Materials science, Metals and alloys

## Abstract

The surface film on pure magnesium and two aluminium-containing magnesium alloys was characterised after 96 h at 95% RH and 22 °C. The concentration of CO_2_ was carefully controlled to be either 0 or 400 ppm. The exposed samples were investigated using X-ray photoelectron spectroscopy, Fourier transform infrared spectroscopy, X-ray diffraction, and electron microscopy. The results showed that when the alloys were exposed to the CO_2_-containing environment, aluminium cations (Al^3+^) was incorporated into a layered surface film comprising a partially “hydrated” MgO layer followed by Mg(OH)_2_, and magnesium hydroxy carbonates. The results indicated that aluminium-containing magnesium alloys exhibited considerably less localised corrosion in humid air than pure magnesium. Localised corrosion in the materials under investigation was attributed to film thinning by a dissolution/precipitation mechanism.

## Introduction

The characteristics of the surface film formed upon magnesium and its alloys are significant in the context of corrosion. When exposed to air (O_2_) or low concentrations of water vapour, magnesium forms a thin (2–3 nm), Cabrera-Mott type oxide (MgO) film, which protects the metal against further oxidation^1^. The chemistry and properties of the thin oxide layer are largely impacted by water (vapour). Many workers have to date employed high-resolution analytical tools to investigate the characteristics of the surface films formed upon magnesium and its alloys after exposure to various environments^2–8^. Examples of the surface-sensitive techniques that have to date provided useful information in this context include transmission electron microscopy (TEM)^2–6^, X-ray photoelectron spectroscopy (XPS)^9,10^, and Auger electron spectroscopy (AES)^8,11–13^.

In the early 1990s, Splinter et al.^14^ utilised XPS, AES, and nuclear reaction analysis (NRA) to examine the water-magnesium interactions at the sub-micron scale. They reported on the formation of MgO with traces of hydrogen at the metal/oxide interface and suggested ‘*it is likely as hydroxy groups trapped in the film*’. Nordlien et al.^15^ used TEM, for the first time, to study the surface film formed on magnesium exposed to air, water (vapour). They observed a ‘dense’ air-formed MgO film, which was transformed into amorphous Mg(OH)_2_ platelets in the presence of water. Nordlien et al.^16,17^ also examined the film formed on magnesium–aluminium alloys and suggested that an ‘alumina component’ was formed in the innermost layer of the film, contributing to alloys’ corrosion performance. More recently, Santamaria et al.^18^ used XPS and photocurrent spectroscopy (PCS) to investigate the surface film formed on magnesium when immersed in liquid water. They reported on the formation of a bilayer film consisting of a thin MgO inner layer and a magnesium hydroxide top layer. Similar results were reported by Taheri et al.^2,3^ who employed FIB and TEM to study the structure and composition of the surface film formed on magnesium after exposure to liquid water and humid air.

In *atmospheric* corrosion, the surface electrolyte, which is sourced by adsorbed or liquid water from the atmosphere, plays a key role in the dissolution process. At a given temperature, the equilibrium amount of adsorbed water on a surface is a function of relative humidity (RH)^1,19,20^. At room temperature, the amount of water at 20% RH corresponds to about one monolayer while about 10 monolayers (~ 3 nm) of water are present at 95% RH^19^. In the latter case, the aqueous layer has liquid-like properties. Hence, to understand the role of the surface electrolyte in the corrosion process under atmospheric conditions (i.e. in the presence of monolayers of electrolyte, deicing salt, and atmospheric pollutants), it is necessary to investigate the corrosion of magnesium alloys under controlled environments and constant RH, see e.g.^21^. At > 90% RH, water molecules partly hydroxylate MgO and heavier tarnish films develop, the principal corrosion product being crystalline brucite; Mg(OH)_2_. This product reacts with atmospheric CO_2_, forming magnesium (hydroxy) carbonates with a slower kinetics^1^. In the context of atmospheric corrosion, Feliu et al.^22,23^ utilised XPS to study the chemistry of the surface film formed on aluminium-bearing alloys (AZ31, AZ80, and AZ91D) exposed in the presence of humid air and CO_2_. They proposed the formation of a layered surface film comprising MgO/Mg(OH)_2_/magnesium carbonate (MgCO_3_). They also suggested that there is relationship between the amount of carbonate-rich film and the subsequent corrosion behavior in humid environment and that a thicker carbonate layer corresponded to slower dissolution rate.

Despite the vast number of publications in the context of magnesium alloy corrosion and the properties of the surface films, there are still some unresolved scientific issues yet to be explored or verified. For example, some researchers have suggested that the Al enrichment at the alloy/oxide interface has a ‘metallic character’^24^, while others believe that the enrichment is owing to the formation of oxidised aluminum (Al^3+^)^8^. In addition, the characteristics of the surface films formed on magnesium and its alloys under anthropogenic (atmospheric) environments are less widely studied as compared with that of aqueous environments. This is because most of the publications cited above relate to full immersion or purely electrochemical experiments. This may not mimic the situation in real outdoor environments, where the corrosion process is greatly influenced by the parameters that are specific to atmospheric corrosion (including 350–400 ppm CO_2_).

In the present study, the film growth and localised corrosion of commercially pure (CP) magnesium and the two Mg–Al alloys AM50 and AZ91 were investigated using well-controlled atmospheric exposures at 95% RH and 22 °C. To provide further insights into the nature of the surface films formed on magnesium and its alloys during atmospheric exposures, the surface film, and corrosion products were carefully studied by a combination of microscopy (FIB and SEM), XPS, X-ray diffraction (XRD) in grazing incidence mode (GI–XRD) and Fourier-transform infrared spectroscopy (FTIR) techniques.

## Experimental

### Test material

Commercially pure magnesium (ingot) and two high purity Mg–Al alloys (AM50 and AZ91) in the high-pressure diecast (HPDC) state (see^25,26^) were used as the test materials, see Table 1 for the chemical composition.

**Table 1:** Alloy composition (wt%).

Material	Al	Zn	Mn	Si	Fe	Cu	Ni	Ca
CP Mg	0.0030	0.0050	0.0023	0.0030	0.0018	0.0003	0.0002	0.0010
AM50	5.0	0.01	0.25	0.01	0.0016	0.0010	0.0007	n.a
AZ91	9.4	0.75	0.18	0.07	0.0074	0.0042	0.0007	0.0005

The HPDC materials were cut to generate 15 × 15 × 3 mm samples. The samples were initially ground on SiC papers (up to P4000 mesh). The samples were polished using 3 and 1 µm diamond suspension, following with a final polishing step using OPS to produce a mirror-like surface. The polished specimens were ultrasonically cleaned dried using a blower (cool air). Next, the samples were then stored in a desiccator over a desiccant for 24 h before the corrosion exposures.

### Exposure set-ups and post-exposure analysis

The atmospheric exposures were performed at a constant temperature (22 ± 0.05 °C) and constant RH of 95 ± 0.5%, lasted for up to 96 h, and the CO_2_ concentration of the exposure gas was carefully controlled to be either 0 and 400 ± 20 pm. In the latter case, the experiment was conducted in flowing air by means of a home-built experimental set-up described elsewhere^1,19,27^. The CO_2_-free corrosion experiments were conducted in a sealed desiccator, wherein a container of a KOH (aq) solution was placed. The function of the KOH (aq) solution was to capture the entire CO_2_ gas inside the desiccator and to maintain the RH of the closed (sealed) environment to be 95.0%, see the details of this experiment in a previous publication^28,29^. The weight of the coupons was measured using a six decimal (0.0001 mg) Sartorius Microbalance in order to register the change in the coupons’ mass as a result of corrosion. Duplicate and triplicate samples were exposed.

In order to study the composition and chemistry of the surface films, XPS measurements were performed by means of a PHI 5500 spectrometer (equipped monochromatic Al-Kα source (1486.6 eV)). The acquisition parameters used for the XPS survey spectra were optimised to be as the following: constant pass energy of 93 eV, a take-off-angle in the range 45–50° (0.38 eV/step). The acquisition conditions for the XPS region spectra included a constant pass energy of 23 eV, a take-off-angle of 45° (0.1 eV/step). For the XPS depth profiling, successive measurements were conducted upon argon (Ar) ion sputtering (4 kV) at an etch rate of 20.3 Å /minute.

The microstructure of the as-cast material and the morphology of the corroded surfaces were investigated by an FEI Quanta 200 scanning electron microscope (SEM). In addition, a Versa 3D DualBeam FIB instrument was utilised to fabricate cross-sections. In-situ Platinum (Pt) deposition was conducted on the specimens’ top surface to minimise the ion milling-induced damage. A decreasing current regime of the ion beam from 42 to 0.24 nA, at the accelerating voltage of 30.00 kV, was used between the rough milling (high current) and fine polishing (low current) steps (see^30,31^). Secondary electron (SE) and backscattering electron (BSE) imaging were performed at the accelerating voltage of 5.00 kV and a working distance of 10.0 mm. The “curtain effect” can be seen in the cross-sectional images prepared in this work. This is an artifact of FIB that can occur while milling a surface with an irregular shape. Thus, in the present work, the relatively thicker corrosion features were skipped from the cross-section preparation.

The structure of crystalline corrosion products formed on the surface of the coupons under different atmospheric conditions was first characterised by means of a Siemens D5000 X-ray diffraction (XRD) system. This system was equipped with a Göbel mirror, CuKα radiation (λ = 1.5418 Å). The XRD data were collected under 2θ: 5–80° and an incidence angle of 0.05° through the Grazing angle incidence arrangement (GI-XRD). Diffraction peaks from the metal substrate were observed in all cases. Thus, the entire corrosion product layer was probed. Finally, Infrared (IR) spectroscopy was utilised to investigate the functional group distributions in the corrosion products influenced by anion transfer. Details of the IR spectroscopy has already been provided in^19^.

## Results

Table 2 presents the average wet and dry mass gains after 96 h at 95% RH. The dry mass gain (measured after 24 h drying over a desiccant at room temperature and ambient pressure) was only a fraction (< 1/4) of the mass gain measured directly after exposure (the wet mass gain). Both in the presence and the absence of CO_2_ the order of the mass gains was: AM50 > CP magnesium > AZ91.

**Table 2:** Average mass gain (wet and dry mass gain*) of CP Mg, AM50 and AZ91 after 96 h exposure at 95% RH and 22 °C, in the presence and absence of CO_2_.

Sample condition	CO_2_ concentration (ppm)	Mass gain (µg/cm^2^)		
		Material		
		CP Mg	AM50	AZ91
Wet	400	70 ± 5	165 ± 41	61 ± 6
	0	52 ± 2	66 ± 7	40 ± 2
Dry	400	15 ± 3	30 ± 8	4 ± 1
	0	12 ± 4	18 ± 6	2 ± 0

*Wet mass gain: Mass gain measured directly after the exposure ends. Dry mass gain: Mass gain measured after 24 h drying over a desiccant at room temperature and ambient pressure.

### Plan view SEM imaging

**Figure 1:**
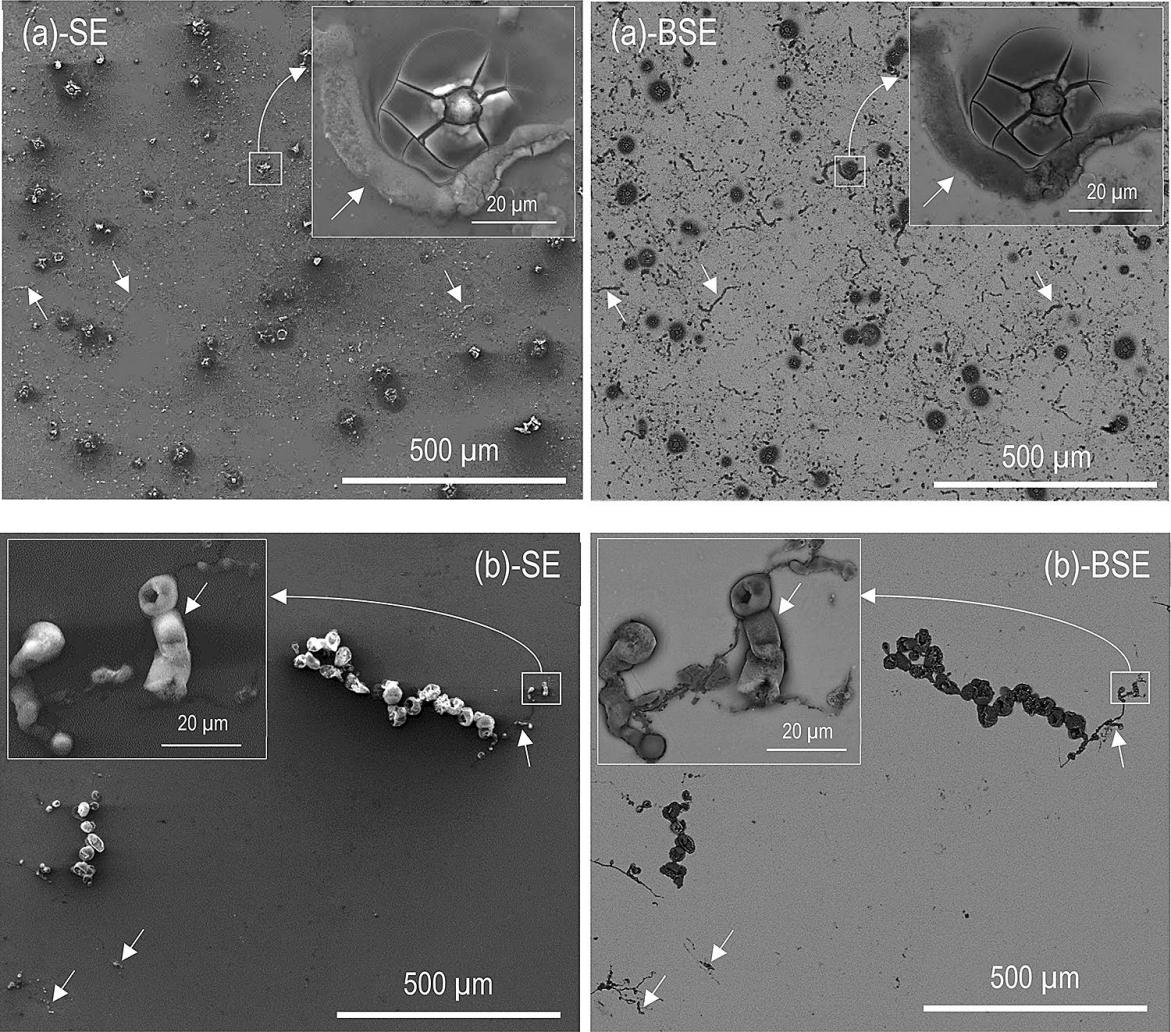
SE and BSE micrographs showing the surface morphology after 96 h exposure at 95% RH and 22 °C. (**a**) CP Mg, 400 ppm CO_2_, (**b**) CP Mg, absence of CO_2_.

**Figure 2:**
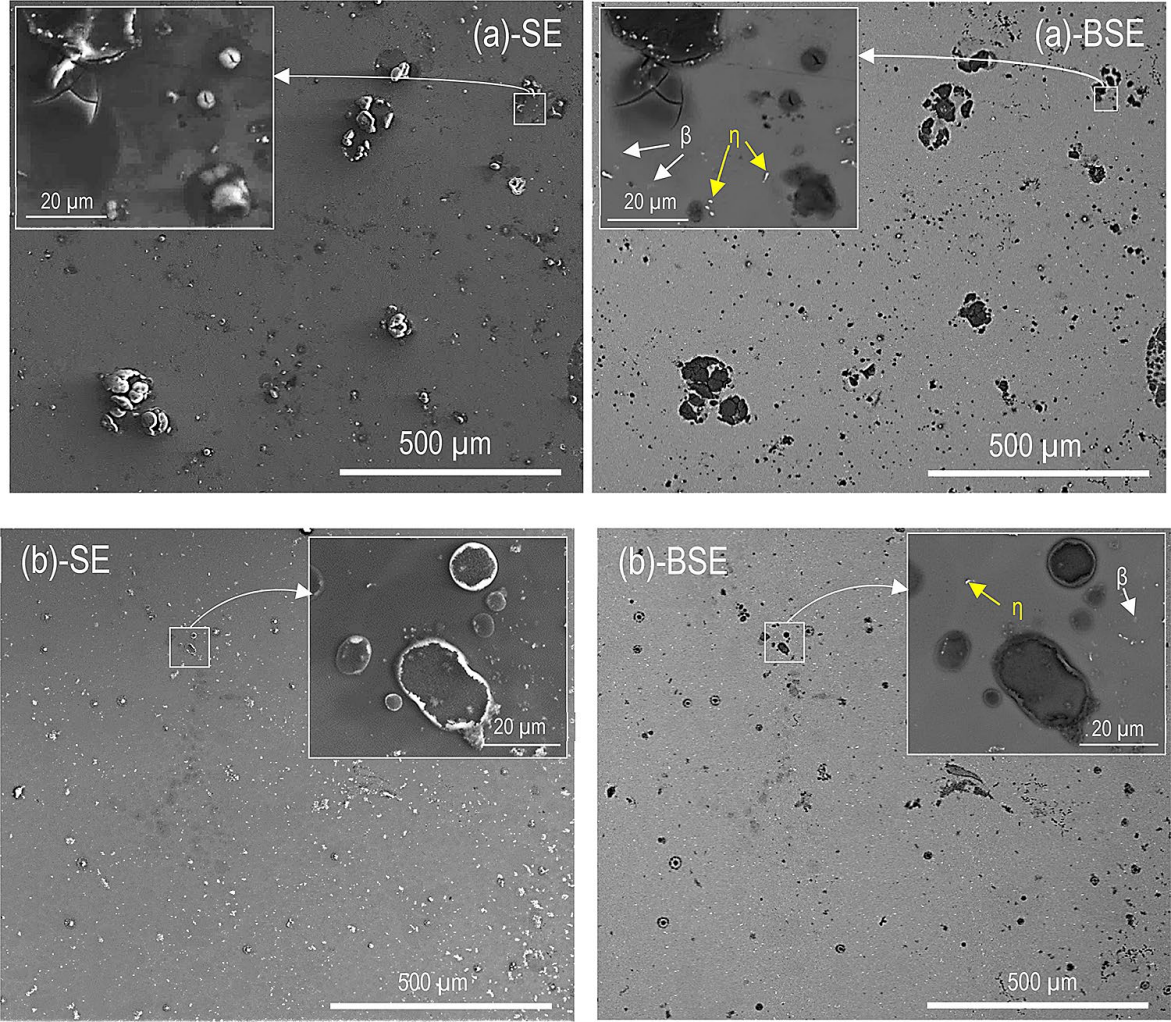
SE and BSE micrographs showing the surface morphology after 96 h exposure at 95% RH and 22 °C. (**a**) AM50, 400 ppm CO_2_, (**b**) AM50, no CO_2_.

**Figure 3:**
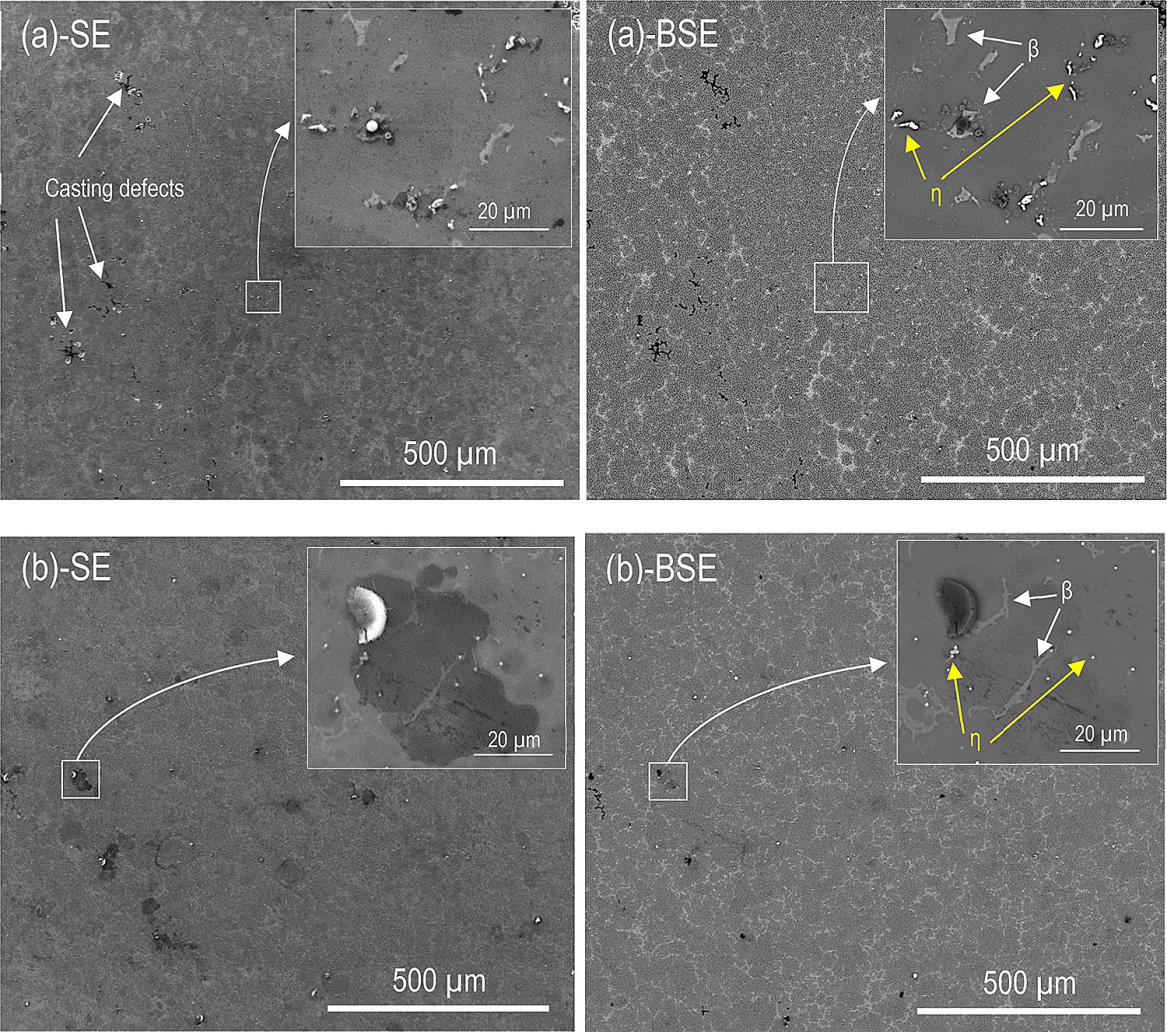
SE and BSE micrographs showing the surface morphology after 96 h exposure at 95% RH and 22 °C. (**a**) AZ91, 400 ppm CO_2_, and (**b**) AZ91, no CO_2_.

The plan view SEM micrographs in Figs. 1, 2, and 3 show the surface morphology of CP Mg, alloy AM50, and alloy AZ91 after 96 h at 95% RH and 22 °C in the presence and the absence of CO_2_. For each case, a secondary electron (SE) and a backscattered electron (BSE) image of the same area is shown. All exposed samples exhibited scattered corrosion product accumulations on the surface. SEM-EDXS showed that the features mainly consisted of magnesium and oxygen. The particles of β- (Mg_17_Al_12_) and η- (Al_8_Mn_5_) phase were detected in the matrix of the alloy samples.

In the presence of CO_2_, CP magnesium (Fig. 1a) formed nodules of different sizes and filaments (see the arrows in Fig. 1a). In some cases, the periphery of the nodules exhibited radial cracks. In the absence of CO_2_, much of the metal surface was seemingly unaffected by corrosion. In this case, the corrosion nodules tended to line up in rows (see Fig. 1b) resulting in filiform corrosion morphologies. However, the tendency for filiform corrosion was not as strong as in the presence of 400 ppm CO_2_ (see arrows in Fig. 1b).

Figure 2 depicts alloy AM50 exposed in the presence and absence of 400 ppm CO_2_, showing corrosion product nodules of different sizes, similar to those observed on CP Mg. However, the filiform corrosion was not observed on alloy AM50. The corrosion product nodules were often associated with η- and β-phase particles in the alloy matrix (see BSE images in Fig. 2). Similar to CP Mg, the corrosion products formed in the presence of CO_2_ contained cracks. The main effect of CO_2_ on the corrosion morphology of alloy AM50 was clusters of large corrosion product nodules that only appeared in the presence of CO_2_ (compare Figs. 1 and 2).

Figure 3 shows that AZ91 was considerably less affected by corrosion than the other two materials, in accordance with the mass gain results in Table 2. The semi-continuous bright network in the BSE micrographs in Fig. 3 corresponds to the β-phase in the alloy.

The corrosion product nodules were similar to those formed on AM50 but they were less frequent and tended to be smaller (compare Figs. 2 and 3). Similar to alloy AM50, the corrosion product nodules tended to occur in the vicinity of η- or β-phase particles. There was no tendency for filiform corrosion.

### Cross-section analysis

**Figure 4:**
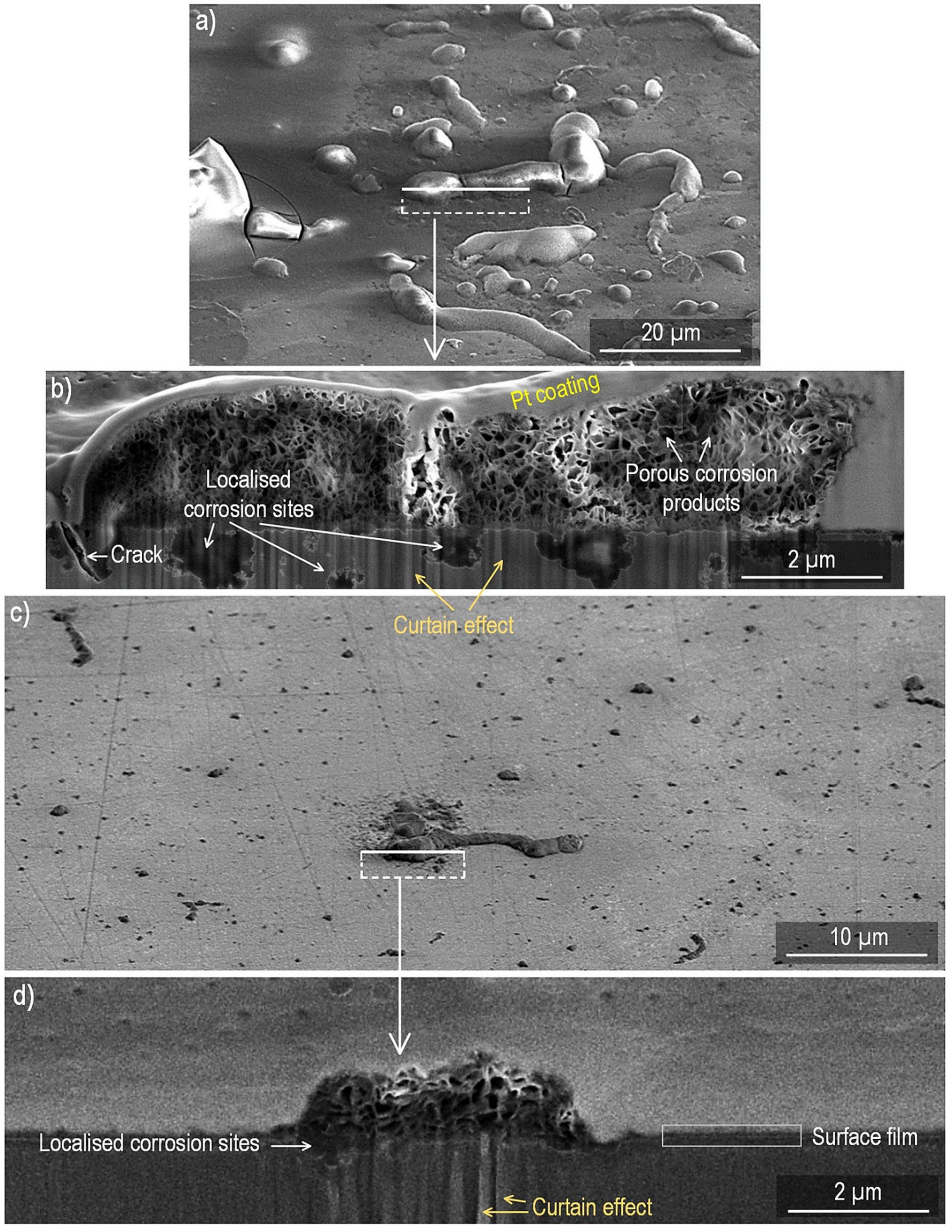
Micrograph of CP Mg exposed at 95% RH and 22 °C for 96 h. (**a**) 52° tilted surface–400 ppm CO_2_. (**b**) Cross–section of the selected area–400 ppm CO_2_. (**c**) 52° tilted surface–no CO_2_. (**d**) Cross–section of the selected area–no CO_2_.

Figure 4 shows cross-section SEM images of CP magnesium exposed for 96 h at 95% RH and 22 °C. The overview image (at 52° tilt angle) in Fig. 4a (400 ppm CO_2_) shows the characteristic filiform corrosion features. Figure 4b presents a cross-section close to the “head” of a filament, (marked in Fig. 4a), showing the internal structure of the filament and the corrosion pits below it. The filament is very porous, consisting of plate-like crystallites. In contrast, the corrosion product within the pits appears denser. At the left edge of the picture; i.e. at the filament’s head, there is an open crack that extends into a corrosion pit.

Figure 4c shows an overview image (at 52° tilt angle) of a CP magnesium sample after exposure in the absence of CO_2_, showing a corrosion product filament at the center. The corresponding cross-section is shown in Fig. 4d, revealing the porous filament interior and the associated corrosion pits in the metal.

**Figure 5:**
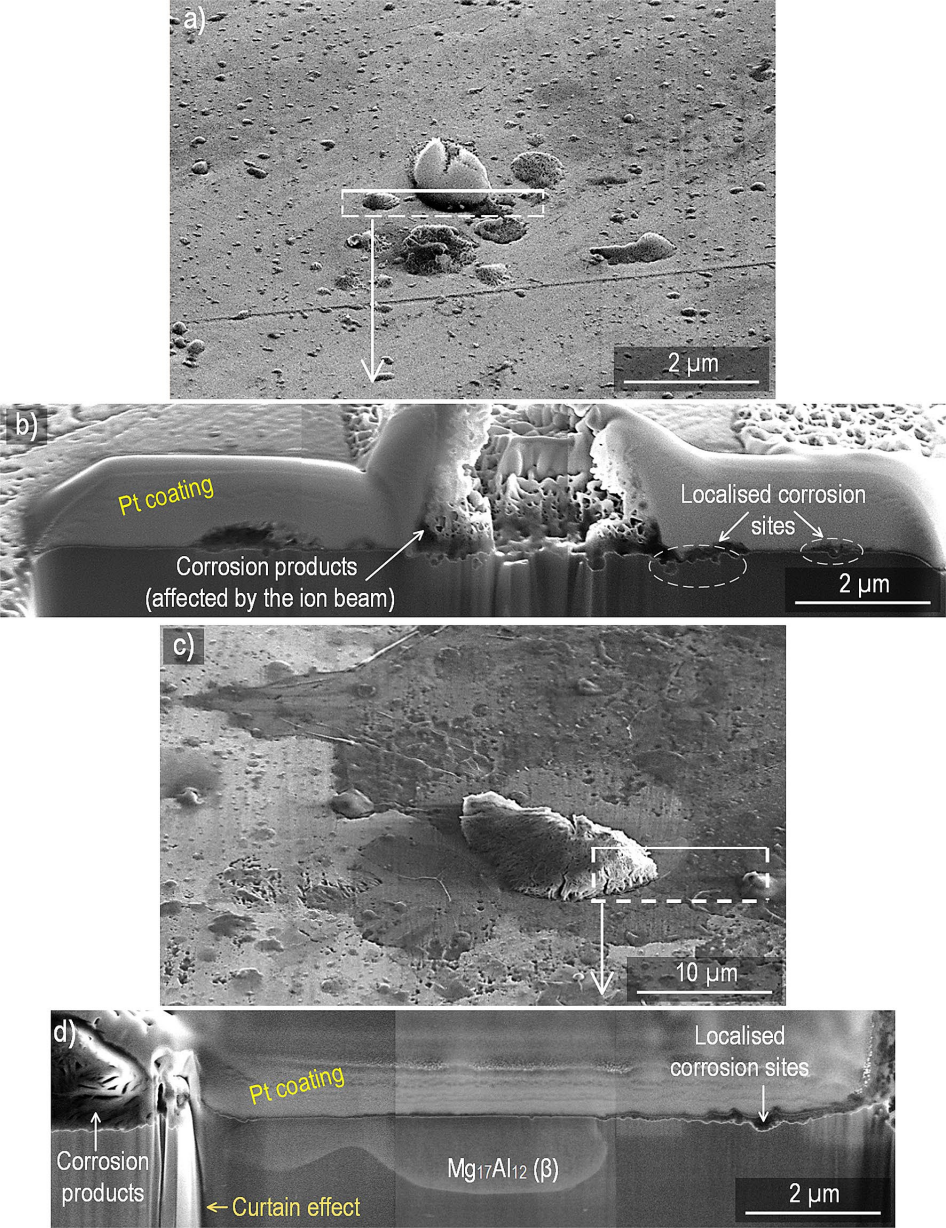
Micrograph of alloy AZ91 exposed at 95% RH and 22 °C for 96 h. (**a**) 52° tilted surface–400 ppm CO_2_. (**b**) Cross–section of the marked area–400 ppm CO_2_. (**c**) 52° tilted surface–no CO_2_. (**d**) Cross–section of the selected area–no CO_2_.

The cross-section images of alloy AZ91 in Fig. 5 shows corrosion product nodules overlying corrosion pits in the alloy. Similar to CP Mg, corrosion product nodules are porous. Although the corrosion product in Fig. 5b was partly destroyed by the ion beam, its plate-like crystallites can still be discerned. Figure 5d shows a β-phase region in the middle of the image with a relatively thick corrosion product on its left side and some localised corrosion on the right side. Alloy AM50 exhibited similar cross-section morphologies (not shown).

### XRD and FTIR analysis

**Figure 6:**
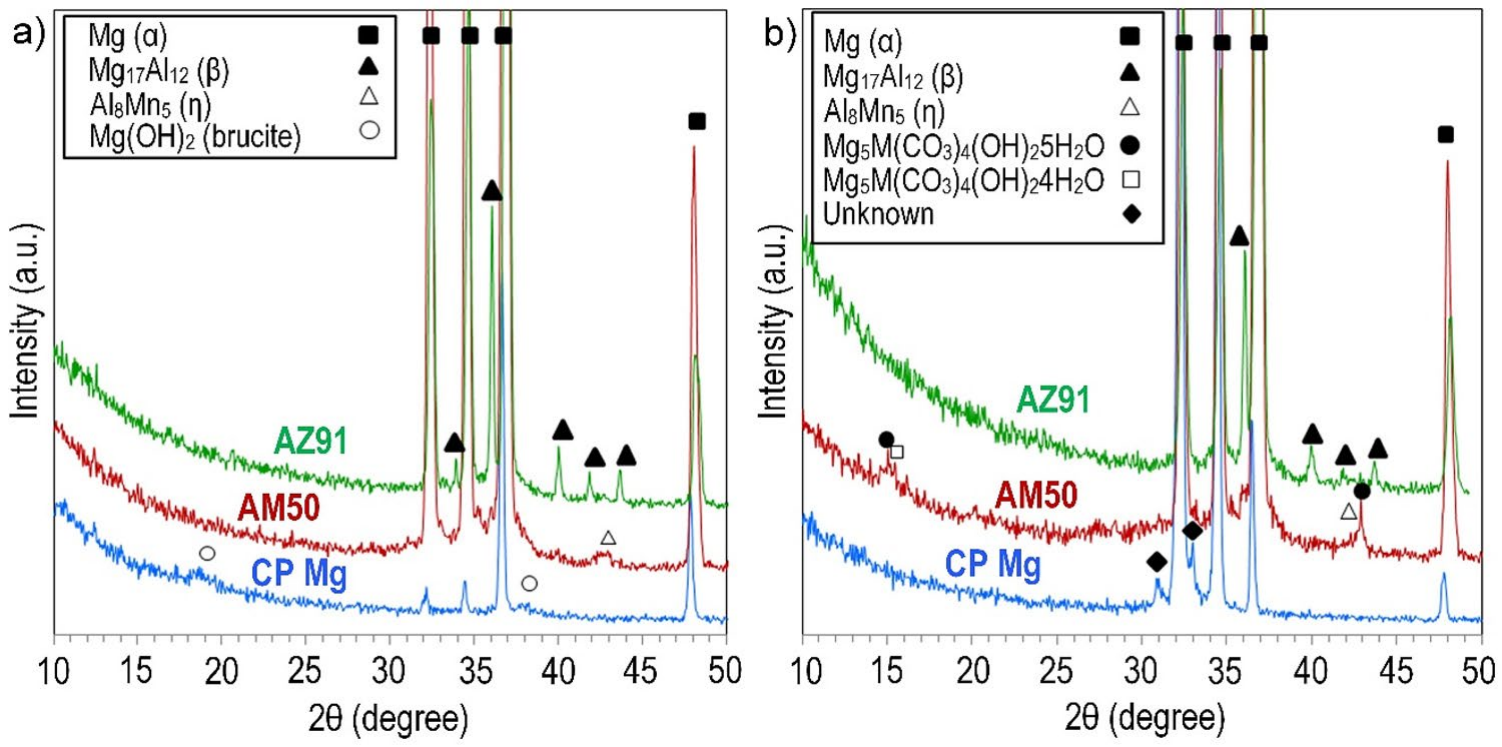
GI–XRD patterns (0.5° incidence angle) for CP Mg (lower (blue) pattern), AM50 (middle (red) pattern) and AZ91 (upper (green) pattern) exposed at 95% RH and 22 °C in the (**a**) absence and (**b**) presence of CO_2_.

Despite a significant mass gain and although corrosion products were observed by SEM (See Figs. 1, 2, 3), no crystalline corrosion product could be identified by XRD using 3° grazing incidence angle. Using a lower incidence angle of 0.5° and longer measuring times to enhance the surface sensitivity (see Fig. 6), brucite (Mg(OH)_2_) was identified on CP magnesium in the absence of CO_2_ (see Fig. 6a). After exposure in the presence of 400 ppm CO_2_, dypingite (Mg_5_(CO_3_)_4_(OH)_2_5H_2_O) was identified on alloy AM50. In addition, a weak peak was observed which was tentatively attributed to hydromagnesite (Mg_5_(CO_3_)_4_(OH)_2_4H_2_O). In the case of CP magnesium exposed in the presence of CO_2_, two peaks were observed which could not be attributed to any compound. No crystalline corrosion products were detected on alloy AZ91.

**Figure 7:**
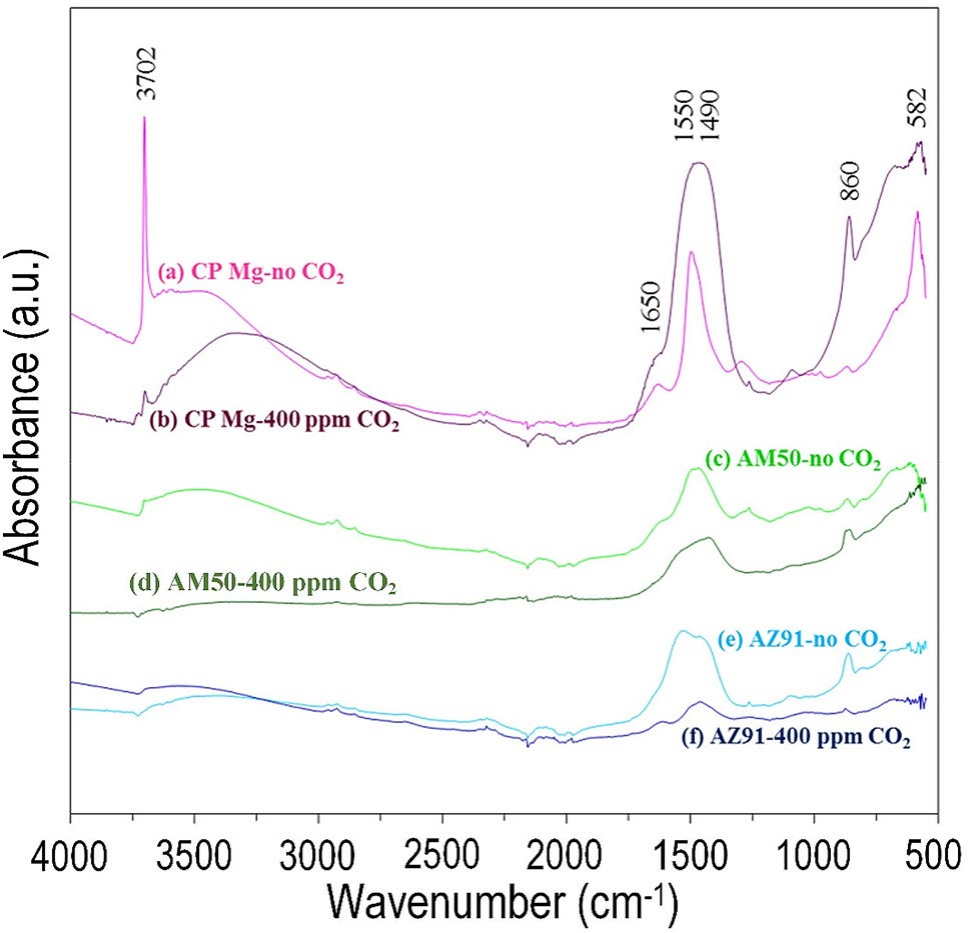
FTIR curves acquired from (**a**) Pink line CP Mg/no CO_2_, (**b**) Violet line CP Mg/400 ppm CO_2_, (**c**) Light green line AM50/no CO_2_, (**d**) Green line AM50/400 ppm CO_2_, (**e**) Light blue line AZ91/no CO_2_ and (**f**) thick blue line AZ91/400 ppm CO_2_ exposed at 95% RH and 22 °C for 96 h.

Figure 7 depicts FTIR spectra from CP Mg, AM50, and AZ91 specimens exposed for 96 h in the presence and absence of atmospheric CO_2_ at ambient temperature and 95% RH. FTIR was used because of the high surface sensitivity. For this measurement, MgO, Mg(OH)_2,_ and a magnesium hydroxy carbonate (Mg_5_(CO_3_)_4_(OH)_2_5H_2_O) powders were utilised as reference materials. It was noted that magnesium exposed in the CO_2_-free environment produced a relatively sharp FTIR peak that is associated with brucite at 3702 cm^-1^. This accords well with the XRD results presented in Fig. 6. It was also noted that magnesium exposed in the CO_2_-containing environment generated a relatively low-intensity peak at the same spot (3702 cm^−1^). The comparatively broad absorption feature from 3600 to 3000 cm^−1^ is linked to O–H stretching vibrations (hydrogen-bonded) in water molecules as well as to hydroxide ions. The fairly weak peak observed in the range 1650 to 1640 cm^−1^ is attributed to water. It is interesting to note that the brucite (Mg(OH)_2_) peak at 3700 cm^−1^ was absent for the alloys. The peaks centered at 1498 cm^−1^ represent the C–O stretching vibrations in carbonate-containing compounds on the sample’s surface. A comparison with the spectra of the two carbonate-containing corrosion products, namely dypingite and hydromagnesite (see e.g.^32^)), implied that all spectra had indications for the formation of magnesium hydroxy carbonate. Therefore, the features at 580, 860, 1490, and 1550 cm^-1^ are ascribed to the compound Mg_5_(CO_3_)_4_(OH)_2_5H_2_O. Finally, it is noted that the carbonate peaks on specimens exposed in the CO_2_-free environment are due to the uptake of carbon dioxide during the FTIR measurement.

### XPS analysis

**Figure 8:**
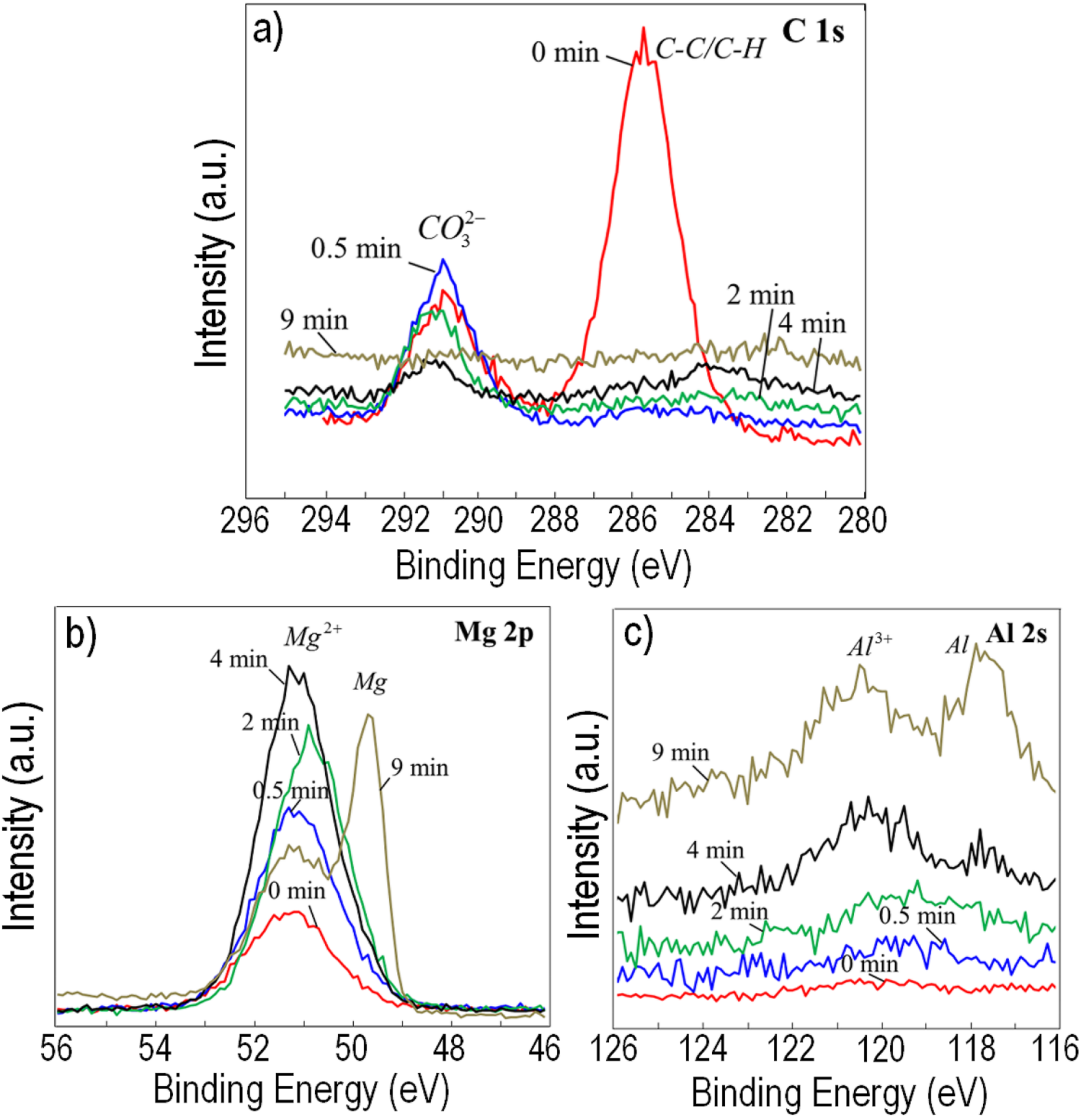
XPS spectra: (**a**) C 1s, (**b**) Mg 2p, (**c**) Al 2s; for AM50 exposed in the 95% RH and 22 °C, in the presence of 400 ppm CO_2_ and in the absence of salt for 96 h.

Figure 8 presents the main three XPS spectra (C 1s, Mg 2p, and Al 2s) acquired from the Mg–Al alloy AM50 after exposure to the CO_2_-containing environment at ambient temperature for 96 h. Carbonate (Fig. 8a) and Mg^2+^ (Fig. 8b) were both identified on top of the surface film. The peaks were linked to the formation of oxide/hydroxide (MgO/Mg(OH)_2_) as well as the carbonate-containing compounds. It was noted that the carbonate peak disappeared only after nine minutes of Ar^+^ sputtering, whereas the peak related to magnesium ions was noticed even after extended etching. Excluding peaks attributed to carbonates, similar compositional variation was recorded for the samples exposed to the CO_2_,-free environment. In addition, as can be seen in Fig. 8c, the peak related to oxidised aluminium (Al^3+^) was detected after four minutes of Ar^+^ etching. It is noted that, at this stage, the peak attributed to metallic aluminium (Al) had a somewhat lower intensity compared to oxidised aluminium (Al^3+^).

**Figure 9:**
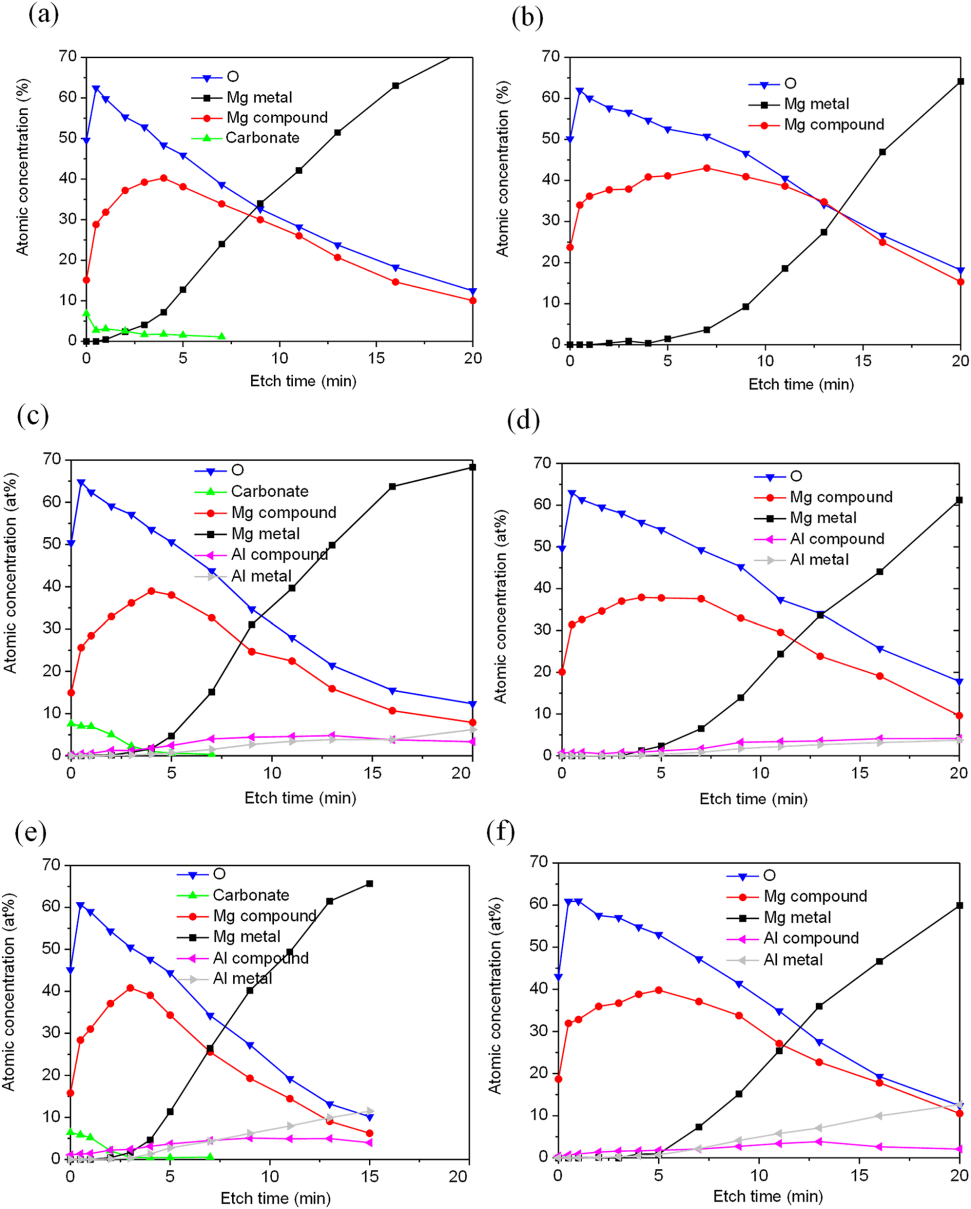
XPS depth profiles for (**a**) CP Mg, (**c**) AM50 and (**e**) AZ91 exposed in the presence and (**b**) CP Mg, (**d**) AM50 and (**f**) AZ91 exposed in the absence of CO_2_ (Adventitious carbon was excluded from the analysis).

Figure 9 shows the XPS depth profiles that were performed on the surface of CP Mg, AM50, and AZ91 after 96 h in the CO_2_-free and CO_2_-containing environments. The recorded XPS C 1s, Mg 2p, and Al 2s photoelectron peaks were curve-fitted using the PHI Multipak software in order to identify the chemical states of these elements. The C 1s component representing adventitious hydrocarbon contamination was excluded from the depth profiles. Carbonate was only detected after exposure to CO_2_ and was confined to a very thin surface layer. The variation of magnesium and aluminium in metallic and ionised forms (Mg^2+^ and Al^3+^) was studied with respect to the etch time. For CP Mg, the atomic fraction of Mg^2+^ at the surface was expectedly somewhat lower in the presence of CO_2_ than in the absence of CO_2_, corresponding to the presence of magnesium hydroxy carbonates and brucite in the presence and the absence of CO_2_, respectively. A similar trend was seen for the two alloys. As the analysis probed deeper strata of the film, the Mg^2+^ signal increased, reaching a maximum of 40–45% at about the depth where the magnesium metal peak appeared.

Alloys AM50 and AZ91 exhibited little Al^3+^ (< 1%) at the surface of the corrosion product layer, as also indicated by the XPS spectra analysis (Fig. 8). A significant Al^3+^ signal only appeared at about the depth where metallic magnesium became prominent, implying that Al^3+^ was enriched at the bottom of the surface film.

In the case of the samples exposed to 400 ppm CO_2_, the signals for metallic magnesium and aluminium appeared after a few minutes of ion etching. The samples exposed in the absence of CO_2_ needed about twice as long etching times to detect metallic signals, indicating that the surface film was thicker in those cases.

**Figure 10:**
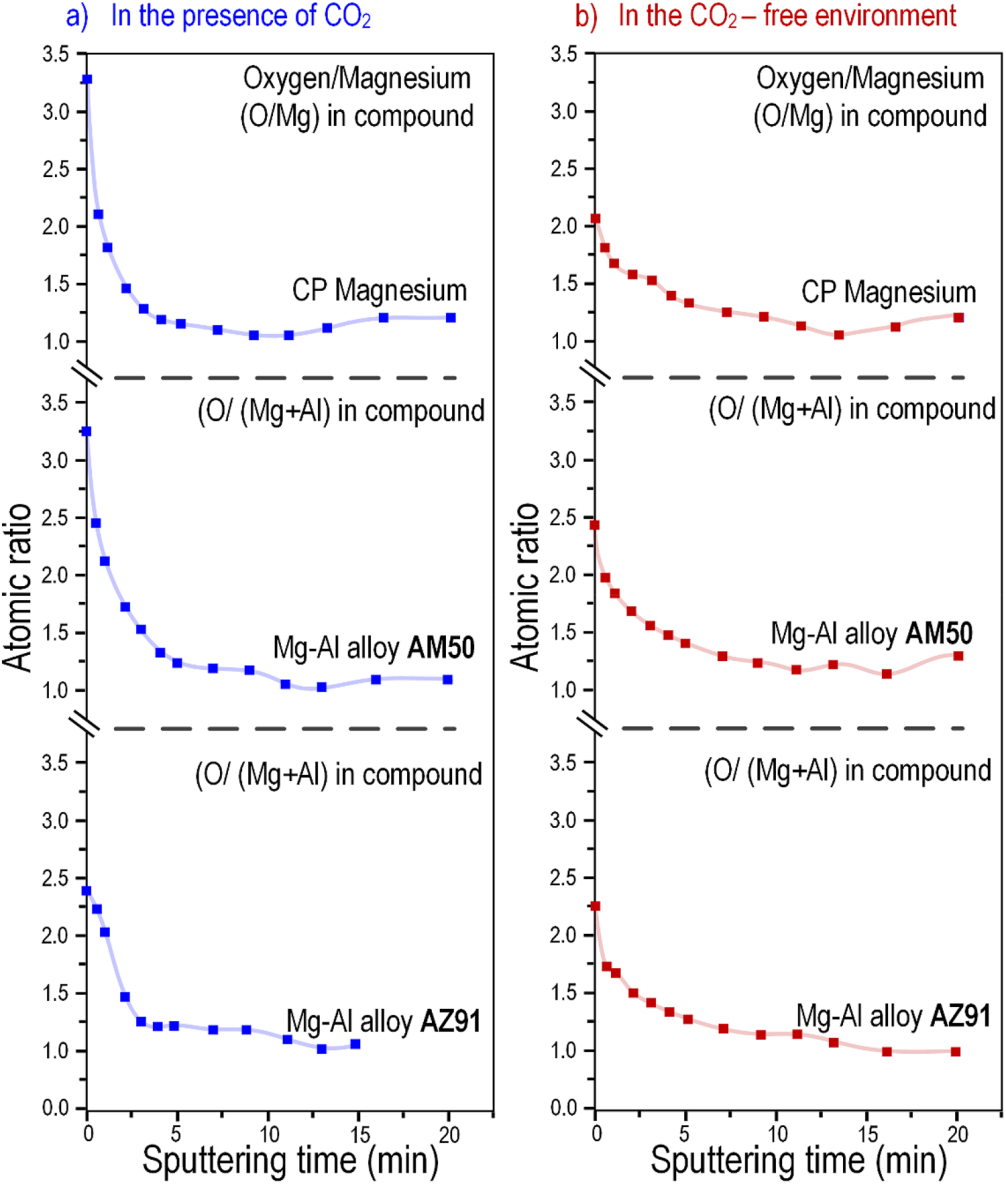
O/Mg^2+^ and O/(Mg^2+^ + Al^3+^) ratios for CP Mg, AM50 and AZ91 after 96 h exposure in 95% RH air (**a**) in the presence of 400 ppm CO_2_ and (**b**) in the absence of CO_2_.

Figure 10 shows the O/Mg^2+^ and O/(Mg^2+^ + Al^3+^) atomic ratios for CP Mg, AM50 and AZ91, in the presence and in the absence of CO_2_, calculated from the XPS data. It may be noted that the O/Mg^2+^ atomic ratio in hydromagnesite is 3.6 while it is 2 in brucite and 1 in MgO. In accordance with the identification of brucite by both XRD and FTIR (see above), the O/Mg^2+^ ratio at the top of the surface film formed on CP magnesium in the absence of CO_2_ was about 2. The observation that alloys AM50 and AZ91 showed O/(Mg^2+^ + Al^3+^) ratios of about 2 after exposure in the absence of CO_2_ indicates that the surface film was dominated by magnesium hydroxide also in those cases.

The higher O/(Mg^2+^ + Al^3+^) ratios detected at the surface in the presence of CO_2_ are attributed to the presence of carbonate (compare with Fig. 10). For all three materials (i.e. CP Mg, AM50 and AZ91) and in both environments (i.e. with 400 ppm CO_2_ and no CO_2_) the O/(Mg^2+^ + Al^3+^) molar ratio decreased rapidly when sputtering started, stabilising at a level slightly above 1. This indicates that the deeper levels of the surface films are dominated by MgO.

## Discussion

### Surface film formation

Because the surface film covers most of the sample surface while the nodules and filaments cover < 10% of the area, the information gathered by the surface-sensitive methods (FTIR, GI–XRD, and XPS) is considered to be dominated by the surface film. After exposure of CP magnesium to the CO_2_–free air, brucite was identified both by GI–XRD (Fig. 6a) and FTIR (Fig. 7). Accordingly, the XPS analysis indicated an O/Mg^2+^ ratio of about 2 in the top part of the corrosion product layer. This observation is in accordance with the studies cited in the introduction section. In contrast, GI-XRD and FTIR analysis of the two alloys in the absence of CO_2_ did not produce evidence for brucite. Still, the XPS analysis showed that the top part of the surface film on the two alloys had an O/Mg^2+^ ratio of about 2 implying that the top part of the surface film on the alloys in the absence of CO_2_ was dominated by X-ray amorphous magnesium hydroxide.

The lack of evidence for brucite on the alloys is attributed to the presence of Al^3+^ in the film (see Figs. 8 and 9 and discussion below). The observation that, for all three materials, the XPS O/Mg^2+^ and XPS O/(Mg^2+^ + Al^3+^) ratios rapidly decrease from 2–2.5 at the surface to a value slightly above 1 implies that the interior of the surface film is dominated by MgO (see Fig. 10b). Table 3 lists corrosion products reported to form on magnesium and on magnesium- aluminium alloys in the atmosphere.

**Table 3:** Corrosion products reported on Mg and MgAl alloys after atmospheric exposures and their respective O/(Mg + Al) ratios.

Corrosion product	O/(Mg + Al)	References
MgO	1	^15,34^
Mg(OH)_2_	2	^44,58^
MgAl_2_(OH)_8_	2.6	^56^
Mg_6_Al_2_(OH)_18_·4.5H_2_O	2.8	^58^
MgCO_3_	3	^21,56^
Mg_5_(CO_3_)_4_(OH)_2_·4H_2_O	3.6	^51,56,58^
(Mg_0.833_Al_0.167_)(OH)_2_(CO_3_)_0.083_·0.75H_2_O	3.7	^61^
Mg_5_(CO_3_)_4_(OH)_2_·5H_2_O	3.8	^21,58^
Mg_2_(OH)_2_CO_3_·3H_2_O	4	^44^
Mg_5_(CO_3_)_4_(OH)_2_·8H_2_O	4.4	^58^
MgCO_3_·3H_2_O	6	^56^

As expected, the materials exposed in the presence of 400 ppm CO_2_ formed surface carbonate, as evidenced by FTIR (Fig. 7) and especially, by XPS (Figs. 8 and 9). Also, magnesium hydroxy carbonate was detected on alloy AM50 by GI-XRD (see Fig. 6 (b)). The relatively high XPS O/Mg^2+^ and XPS O/(Mg^2+^ + Al^3+^) ratios at the surface (see the ratios for CP Mg and AM50 in Fig. 10a) are in accordance with the presence of carbonate. Similar to the samples exposed in the absence of CO_2_, the XPS O/Mg^2+^ and XPS O/(Mg^2+^ + Al^3+^) ratios of the samples exposed in the 400 ppm CO_2_ rapidly decreased upon ion etching, reaching a value slightly above 1 (see Fig. 10a). This indicates that the interior of the surface film is dominated by MgO also in these cases.

The present results thus show that when pure magnesium is exposed to humid CO_2_–free air, it forms a surface film dominated by MgO and having a magnesium hydroxide top layer. The surface films formed on the two alloys are similar except that (*i*) Al^3+^ is also incorporated into the film, the aluminium concentration being highest at the alloy/oxide interface (see Fig. 8b, the ratios for AM50 and AZ91), and (*ii*) the hydroxide layer is amorphous. It may be noted that although alloy AZ91 contains 1% (by weight) of zinc, Zn did not show up in the XPS depth profiles of the surface film. There was also no Zn enrichment at the metal/film interface as reported for AZ31B after immersion in aqueous solution^33^. Exposure in the presence of 400 ppm CO_2_ produced very similar films, except that they also featured a thin surface layer of carbonate.

The observation that the XPS O/Mg^2+^ and O/(Mg^2+^ + Al^3+^) ratios within the film are slightly higher than 1 (see Fig. 10) is attributed to the presence of magnesium hydroxide in the bulk of the film. In the case of the alloys, the presence of Al^3+^ in the film also tends to increase the XPS O/(Mg^2+^ + Al^3+^) ratio, but this is a minor effect. The presence of hydroxide in the film is in accordance with^34^ who reported that the bulk of the film formed on magnesium is made up of hydrated nano-crystalline MgO. Also, using SIMS to analyze the magnesium surface after exposure in pure water, Unocic et al.^35^ reported that MgO in the film was partly hydrated, the degree of hydration decreasing towards the alloy/film interface. The coexistence of MgO and Mg(OH)_2_ in the surface films is in accordance with the observation that the thermodynamically favored conversion of crystalline MgO to Mg(OH)_2_ (brucite) under ambient conditions is slow^36,37^. Similarly, Lee et al.^38^ report that when exposed to 80% RH N_2_ at ambient temperature, MgO films react to form hydroxide at the surface while the conversion within the film is only partial.

The surface of MgO is indeed known to have a high affinity for water, reacting to form surface hydroxide even at very low partial pressures of water^39^:

1 $$ {\text{MgO(surf)}} + {\text{H}}_{{2}} {\text{O(g)}} \to {\text{Mg(OH)}}_{{2}} {\text{(surf)}} $$

According to Allen et al.^40^, the thermodynamics of hydroxylation are especially favorable for the (111) MgO surface but the formation of surface hydroxide is also favored on other crystal faces at ambient concentrations of water vapor.

Recent experimental and theoretical studies have demonstrated that: (*i*) MgO is nano-crystalline (i.e. it contains a comparatively high density of grain boundaries (GBs) (see e.g.^34^), and (ii) oxide GBs, in cases where there is a high density of them, may function as fast-diffusion channels for water molecules (see e.g.^31,41^). Based on (i) and (ii), it is reasonable to suggest that water can react at GBs in the continuous, polycrystalline MgO film. This process hydroxylates the GBs through a reaction similar to (Eq. 1) and explains the partial hydroxylation of the MgO–part of the film, as implied by the XPS results (Figs. 9 and 10) and previously described by^34,35,42^ (i.e. the hydrated MgO). This process is expected to result in a slowly growing “hydrated” MgO film of relatively even thickness, as reported by Do et al.^43^. The rate of corrosion would be limited by the transport of water across the MgO film. Also, the proposed reaction is expected to generate a gradient in the activity of water across the “hydrated” MgO layer, in accordance with reports in the literature^35^. Because the process depends on hydroxylation of the MgO GBs it is expected to depend on the activity of water in the environment; i.e. on RH. It may be noted that a somewhat similar non-electrochemical corrosion mechanism, in this case involving direct reaction of magnesium and water forming MgO and H_2_(g), has been suggested by Cano et al. for alloy AZ31B under immersion conditions^33^.

### Initiation of localised corrosion

This study shows that in parallel to film growth localised corrosion occurs during exposure to 95% RH air, especially in the case of magnesium and alloy AM50. Localised corrosion was not reported by Do et al.^43^ who exposed magnesium in the air at 50–60% RH. The present observations are in accordance with Lindström et al.^44^ who investigated the initial stages of corrosion of magnesium in humid air and reported a localised attack which was attributed to an electrochemical corrosion process involving anodic dissolution of magnesium and H_2_ evolution. Similar to^44^, localised corrosion of magnesium is suggested to result in brucite formation and the entangled crystal mass seen in Fig. 4 (b) and (d), is hence interpreted as brucite.

Localised corrosion has also been reported to occur on magnesium during immersion in liquid water^1,34^. During immersion the MgO/Mg(OH)_2_ film present on the surface is expected to dissolve:

2 $$ {\text{Mg(OH)}}_{{2}} {\text{(surf)}} \to {\text{Mg}}^{{{2} + }} {\text{(aq)}} + {\text{2OH}}^{ - } {\text{(aq)}} $$

The resulting MgO/Mg(OH)_2_ film thinning is expected to cause corrosion, by increasing the film’s permeability to water, resulting in an acceleration of the direct reaction of water and magnesium at the film/metal interface forming MgO and hydrogen. Also, film thinning may allow electrochemical corrosion to start, including anodic dissolution of magnesium and cathodic reduction of water on adjacent (thin) parts of the film. Under high RH conditions at room temperature, pure metal surfaces are known to be covered by relatively thick layers of adsorbed water. Thus, the equilibrium amount of adsorbed water on a clean surface in the experimental conditions of this paper (95% RH, 22 °C) is reported to be on the order 10 monolayers (~ 3 nm) ^45^. Because these layers have liquid-like properties many of the concepts that apply in aqueous solutions can be used. Of course, there are also many important differences between the corrosion of metal surfaces covered by a thin layer of adsorbed water and corrosion under immersion conditions. Thus, in the present case, localised corrosion cannot be explained by the dissolution of the MgO/Mg(OH)_2_ film in a large volume of water because the adsorbed water film has a very small volume, meaning that dissolution of the film to form a saturated aqueous solution on the surface cannot cause significant film thinning. However, it may be noted that MgO is significantly more soluble than brucite. Thus, K_sp_ MgO = 1 × 10^–6.33^ while K_sp_ Mg(OH)_2_ (brucite) = 1 × 10^–11.16^. Also, the hydrated solid MgO (i.e. Mg(OH)_2_) is not identical to brucite ^34^. Thus, according to Grauer^46^, the magnesium hydroxide surface layer resulting from the hydration of solid MgO is significantly more soluble than brucite with K_sp =_ 1 × 10^–9.2^. This implies that an aqueous solution which is *saturated* with respect to the MgO/Mg(OH)_2_ film is *super-saturated* with respect to brucite. Hence, it is suggested that, at high RH, the MgO/Mg(OH)_2_ film is thinned because it is dissolved in the liquid-like aqueous layer (see the reaction (4)) and re-precipitated as brucite:

3 $$ {\text{Mg}}^{{{2} + }} {\text{(aq)}} + {\text{2OH}}^{ - } {\text{(aq)}} \to {\text{Mg(OH)}}_{{2}} {\text{(brucite)}} $$

Thus, it is suggested that the brucite detected on magnesium in the absence of CO_2_ has formed by reactions (2–3). It has been reported in the literature^47–49^ that MgO is dissolved preferentially by water at sites where the coordination of Mg^2+^ is low (i.e. < fivefold). It may be noted that when precipitated from solution, brucite forms discrete crystallites rather than a film and that brucite formed by this reaction is therefore not expected to re–passivate the surface. Also, in this scenario, MgO film dissolution is expected to be faster on the parts of the surface where brucite nuclei have already formed. This would tend to cause film breakdown to become localised, before the onset of electrochemical corrosion. It may be noted that the high solubility of the oxide compared to the hydroxide is peculiar to magnesium among the more common metallic materials and that the scenario outlined above is hence not applicable to the atmospheric corrosion of metals in general.

The dissolution–reprecipitation mechanism for initiating localised corrosion described above is suggested to apply to magnesium and for the two alloys. It is proposed that electrochemical localised corrosion ensues subsequent to the pit initiation process just described. For the two alloys there is a tendency for the pits to be associated with η and/or β intermetallic particles (see Fig. 5d). This is attributed to the intermetallic particles being cathodic towards magnesium^50^.

Figures 1, 2, and 3 show that filiform-like corrosion only occurred on magnesium and not on the two alloys. Most workers agree that filiform corrosion of uncoated metals is electrochemical and related to the breakdown of passivity. Also, it is considered that the filament head is anodic while the tail and the un-corroded periphery around the head are cathodically active. Filiform-like corrosion of uncoated magnesium and magnesium alloys has been reported by several workers^26,33,51–53^. The close relation of filiform corrosion to passivity is in accordance with^53^ who reports that adding dichromate to an NaCl (aq) solution both improved the protective properties of the surface film on magnesium and caused filiform-like corrosion. In contrast to the present observations, Ghali^54^ claims that filiform corrosion only affects Mg–Al alloys and not pure Mg. Also, Lunder et al.^55^ reported filiform-like corrosion of alloy AZ91 in 3–5% NaCl (aq) solution and argued that the presence of an Al-containing surface film played an essential role for filiform corrosion to occur. Recently, it was proposed that AlMn intermetallics embedded in the filament causes cathodic activation of the corrosion filaments on alloy AZ31B in NaCl (aq) solution^33^. The present observation of filiform corrosion on CP magnesium but not on the two alloys disproves the idea that alloying elements such as Al or the presence of intermetallic inclusions are necessary for filiform corrosion of Mg. In the present case, filiform corrosion was always accompanied by localised corrosion, suggesting that the two phenomena are related. Thus, filiform corrosion is considered to be the result of the movement of electrochemical corrosion cells on the surface while localised corrosion corresponds to stationary corrosion cells. Why the corrosion cells on magnesium move in some cases and not in others is considered to be an open question.

### The effect of Al alloying

This study shows that alloy AZ91 exhibited significantly less localised corrosion in humid air than alloy AM50 and CP magnesium (see Figs. 1–3). According to Nordlien et al.^16^ and Esmaily et al.^8^, increasing the Al content of Mg–Al alloys resulted in a significant increase in the protective properties of the surface film. They proposed that the improved corrosion resistance was due to the presence of a continuous skeletal barrier of alumina in the film. Moreover, Danaie et al. reported^24^ that exposure of alloy AM50 in NaCl (aq) solution resulted in the formation of a thin protective Al-rich layer at the interface between metal and surface film. However, this layer was only observed in regions with high Al content (segregation bands) and not at the center of the α-Mg grains where Al content was low. They suggested that the layer consisted of ''highly defective Al_2_O_3_ with metallic character''. In the present case, analysis by XPS demonstrated the presence of small amounts of Al^3+^ in the surface film on alloys AM50 and the AZ91, the concentration of aluminium increasing towards the bottom of the film (see Fig. 9). Accordingly, it is suggested that the slow corrosion of alloy AZ91 is due to its relatively high Al content and that the presence of aluminium in the surface film enhances its ability to protect against corrosion.

Because MgO does not dissolve alumina except at very high temperatures it is suggested that (when oxidised) aluminium accumulates between MgO grains in the form of Al_2_O_3_ or perhaps spinel; MgAl_2_O_4_. Thus, the corrosion protection effect of aluminium is tentatively attributed to the presence of alumina in the MgO grain boundaries, somewhat similar to the suggestion by Nordlien et al.^15^. The beneficial effects of Al alloying on the atmospheric corrosion of magnesium have been reported in the presence of NaCl^1,56^. In those cases, the effect was attributed to the lower solubility of the alumina component in the passive films at the approximately neutral pH expected at anodic sites. In the present case where the only available electrolyte is water adsorbed on the surface, large pH gradients are unlikely. Instead, the effect of alumina is suggested to be related to the depassivation process described above.

The lack of evidence for brucite on alloys AM50 and AZ91 after exposure in the absence of CO_2_ was already mentioned. It is tentatively attributed to the presence of aluminium in the surface film (see Figs. 8 and 9). Thus, partial substitution of Al^3+^ for Mg^2+^ in the layered brucite structure results in the formation of Mg–Al-layered double hydroxides (LDHs) with anions intercalated between the positively charged brucite-like layers^8,12,57^. Indeed, the LDH compound meixnerite (Mg_6_Al_2_(OH)_18_·4H_2_O), also reported as Mg_6_Al_2_(OH)_18_·4.5H_2_O) has been identified as a corrosion product on Mg–Al alloys in the presence of NaCl^58^. In the present case, it is suggested that a small amount of Al^3+^ substitution causes a broadening of the brucite X-ray diffraction peaks, in accordance with the report of^59^, making identification more difficult. Also, Al^3+^ substitution results in hydrogen bonding of the hydroxide ions, the corresponding changes of the IR spectrum explaining the absence of evidence for brucite by FTIR. The presence of LDH compounds on the surface has been reported to provide protection against corrosion of magnesium alloys. Thus the formation of LDH precipitates on alloy AZ31 resulted in corrosion protection in NaCl (aq) solution^59^ The corrosion protection has been attributed to a a variety of reasons, including ion-exchange, competitive adsorption of chloride ions, and protective deposition of Mg(OH)_2_ on the alloy surface^1,12,60^. The present results do not allow us to conclude whether LDH compounds play a significant role in the corrosion of the two Mg–Al alloys studied.

### The effect of CO_2_

 The present results, showing that a thin carbonate-containing surface layer forms on top of the MgO/Mg(OH)_2_ film in the presence of 400 ppm CO_2_ are in accordance with several reports in the literature. Thus, it has been reported that the MgO/Mg(OH)_2_ films developed by magnesium at 50 °C and 98% RH air^22^ and in immersion experiments allowing CO_2_ access^9,35^ exhibit a carbonate-containing top layer. It has been shown that dissolved CO_2_ reacts to form magnesium hydroxy carbonates on the corroding magnesium surface^44^. Also, carbonate-containing LDHs have been reported to form on magnesium aluminium alloys in the presence of ambient levels of CO_2_ at 66% RH^61^.

The XPS results in Fig. 9 show that for all three materials, the etch time needed to detect metallic magnesium was significantly shorter for the samples exposed in the presence of CO_2_. Thus, it is concluded that the surface film formed was thinner after exposure in the presence of CO_2_ compared to when CO_2_ was absent. This implies that the growth of the MgO/Mg(OH)_2_ film in the presence of water vapor is impeded by CO_2_. Above, it was suggested that the rate of film growth in the presence of water vapour is limited by inward transport of water via surface hydroxylation of MgO. This implies that film growth may slow due to other species that compete with water for adsorption on MgO. Indeed, the paper by Allen et al.^40^ reports that there is a competition between the reactive dissociative adsorption of H_2_O and chemisorption of CO_2_ for some MgO surfaces. Hence, it is hypothesised that the slower growth of the MgO/Mg(OH)_2_ film in the presence of CO_2_ is due to adsorbed carbonate on the film surface and arguably in the MgO grain boundaries that restrict the inward transport of water through the film.

In the scenario described above for localised corrosion of magnesium at high RH (see above), CO_2_ is expected to play a role due to its acidity and because it forms carbonate ions that may form precipitates.

4 $$ {\text{CO}}_{{2}} {\text{(aq)}} + {\text{2OH}}^{ - } {\text{(aq)}} \to {\text{CO}}_{{3}}^{{{2} - }} {\text{(aq)}} + {\text{H}}_{{2}} {\text{O}} $$

Acidification of the adsorbed aqueous layer by CO_2_ is expected to speed up the dissolution of the MgO/Mg(OH)_2_ surface film (see the reaction (2)). Hence, the presence of CO_2_ is expected to initiate localised corrosion at high RH. Accordingly, the present results show that corrosion filaments and nodules were more frequent in the presence of CO_2_ (see e.g. Figure 1). Also, Lindström et al.^44^ reported that the presence of 350 ppm CO_2_ in the air at 95% RH caused a faster onset of magnesium localised corrosion compared to CO_2_–free conditions. As mentioned above, the oxide/hydroxide film tends to be thinner in the presence of CO_2_ (Fig. 10). This is also expected to make the materials more susceptible to (localised) corrosion. It may be noted that CO_2_ tends to slow down corrosion after long exposure times. This effect has been attributed to the formation of a protective layer of magnesium hydroxy carbonate^1,12,17^.

Overall, carefully controlled atmospheric corrosion exposures combined with post-exposure analysis revealed that the surface film formed on magnesium and Mg–Al alloys consisted of a MgO bottom layer and a thin brucite (Mg(OH)_2_) top layer. In the presence of atmospheric CO_2_, the film surface was clearly carbonated. Based on XPS, XRD, and FTIR results and the relevant findings presented in the literature, a phenomenology for the growth of the surface film on magnesium was suggested where water is transported across the MgO layer via hydroxylated MgO grain boundaries. In this scenario, Mg(OH)_2_ in the grain boundaries reacts with magnesium at the film/metal interface, forming fresh MgO and releasing hydrogen. Localised corrosion observed on magnesium and the alloys studied herein was suggested as being initiated by film thinning through a dissolution–precipitation mechanism where the more soluble MgO is dissolved into the liquid-like surface water layer, to be precipitated as the less soluble brucite (or presumably as magnesium-bearing layered double hydroxides–like compounds). The surface films formed on the two Mg–Al alloys (AM50 and AZ91) were similar to the films on commercially pure magnesium, with the exception that contained a few percent (in the range 1–2 at.%) Al^3+^, the aluminium concentration increasing towards the film/alloy interface. It was also noted that, at the very early stages of corrosion (< 100 h), a higher density of localised corrosion sites was observed in the presence of CO_2_. This is attributed to the acidity of CO_2_ that speeds up film dissolution and to the notion that CO_2_ promotes film thinning.

## Data Availability

The raw/processed data required to reproduce these findings cannot be shared at this time as the data also forms part of an ongoing study.
